# Krill Oil Mitigates Cisplatin-Induced Ovarian Toxicity via Attenuation of Oxidative Stress and Inflammatory Pathways

**DOI:** 10.3390/cimb47090708

**Published:** 2025-09-01

**Authors:** Erson Aksu, Oytun Erbas

**Affiliations:** 1 Gynecology and Obstetrics Clinic, Corlu Vatan Hospital, Tekirdağ 59000, Türkiye; 2 Faculty of Medicine, Biruni Research Center, Biruni University, Istanbul 34000, Türkiye; oytunerbas2012@gmail.com

**Keywords:** cisplatin, krill oil, ovarian toxicity, oxidative stress, inflammation, fertility preservation, Nrf2, NLRP3

## Abstract

Cisplatin remains a cornerstone chemotherapeutic agent; however, its off-target gonadotoxicity poses a significant risk for premature ovarian failure (POF) and infertility in young women. Strategies to preserve ovarian function during chemotherapy are critically needed. To investigate the protective effects of krill oil supplementation against cisplatin-induced ovarian damage in a rat model, with a focus on oxidative stress, inflammation, follicular dynamics, and stromal fibrosis. Twenty-one adult female Wistar albino rats were randomized into three groups: control, cisplatin-treated, and cisplatin + krill oil-treated. Ovarian toxicity was induced via intraperitoneal injection of cisplatin (2.5 mg/kg, twice weekly for four weeks). Krill oil (4 mL/kg/day) was administered orally during the same period. Ovarian histopathology, follicle counts (primordial, primary, secondary, tertiary), stromal fibrosis, and biochemical markers, including plasma anti-Müllerian hormone (AMH), malondialdehyde (MDA), tumor necrosis factor-alpha (TNF-α), interleukin-1 beta (IL-1β), and ovarian levels of nuclear factor erythroid 2-related factor 2 (Nrf2), Toll-like receptor 4 (TLR4), TNF-α, NOD-like receptor family pyrin domain containing 3 (NLRP3), and IL-1β were evaluated. Cisplatin significantly reduced primordial, primary, secondary, and tertiary follicle counts while increasing stromal fibrosis (*p* < 0.001). Krill oil co-treatment notably ameliorated follicular depletion—improving follicle counts by 38.16%, 54.74%, 62.5%, 40.43%, respectively—and reduced fibrosis (*p* = 0.017). Biochemically, cisplatin decreased AMH levels and Nrf2 expression while elevating MDA, TNF-α, TLR4, NLRP3, and IL-1β levels (*p* < 0.001). Krill oil supplementation restored AMH (*p* = 0.002) and Nrf2 (*p* = 0.003) levels, while reducing MDA (*p* = 0.009), NLRP3 (*p* < 0.001), ovarian IL-1β (*p* = 0.005), plasma IL-1β (*p* < 0.001), TLR4 (*p* = 0.001), plasma TNF-α (*p* = 0.001), and ovarian TNF-α (*p* < 0.001), compared to the cisplatin group. Krill oil exerts significant antioxidant and anti-inflammatory effects, offering a promising strategy to mitigate cisplatin-induced ovarian damage and preserve fertility in young cancer patients.

## 1. Introduction

Globally, approximately 1,300,196 new cancer cases were reported among individuals aged 15–39 years in 2022, with a notable increasing trend in cancer incidence observed among young adults in recent years [1]. While advancements in oncological treatments have improved survival rates, they have also brought significant iatrogenic challenges, particularly with regard to fertility preservation. Chemotherapeutic regimens, although effective against malignancies, can inflict irreversible damage on the reproductive system, leading to premature ovarian failure (POF) and infertility [2,3]. Given the profound impact on reproductive potential, the incorporation of fertility preservation measures has become a critical consideration in the multidisciplinary management of young female cancer patients.

Cisplatin (cis-diamminedichloroplatinum II) remains a cornerstone chemotherapeutic agent due to its potent antineoplastic activity across a broad spectrum of solid tumors [4]. Its primary cytotoxic mechanism involves DNA adduct formation, which interferes with replication and transcription, ultimately triggering apoptosis [5,6]. However, cisplatin’s non-selective action extends toxicity to healthy tissues, including the ovaries, where it disrupts mitochondrial function, induces oxidative stress, and initiates inflammatory cascades [7,8,9]. Recent studies have shown that cisplatin activates multiple inflammatory pathways, including the upregulation of tumor necrosis factor-alpha (TNF-α), stimulation of Toll-like receptor 4 (TLR4)-mediated signaling, and activation of the NOD-like receptor family pyrin domain containing 3 (NLRP3) inflammasome [10]. TNF-α promotes inflammation and apoptotic signaling [11], while TLR4, acting upstream through the nuclear factor kappa B (NF-κB) pathway, regulates both TNF-α expression and inflammasome activation [10]. NLRP3 activation leads to caspase-1-mediated maturation of interleukin-1 beta (IL-1β) and interleukin-18 (IL-18), further exacerbating tissue injury [12]. In addition to inflammation, oxidative stress also plays a pivotal role, as cisplatin elevates malondialdehyde (MDA) levels and suppresses nuclear factor erythroid 2-related factor 2 (Nrf2), impairing cellular antioxidant defenses [8]. Collectively, the combined activation of inflammatory and oxidative stress pathways contributes significantly to cisplatin-induced ovarian follicle damage, diminished ovarian reserve, and compromised reproductive function [8,9].

Cisplatin is known to induce ovarian injury through oxidative stress and inflammation, ultimately leading to premature follicular loss and infertility [13,14]. Several antioxidant agents, such as melatonin and resveratrol, have shown promise in experimental models; however, their clinical translation remains limited due to drawbacks such as melatonin’s hormonal effects or resveratrol’s poor bioavailability [5,14,15,16].

Krill oil, extracted from *Euphausia superba*, has emerged as a promising alternative due to its distinct biochemical composition. Unlike traditional fish oil, krill oil delivers omega-3 fatty acids (EPA and DHA) primarily in phospholipid form, which enhances bioavailability and tissue uptake [17,18]. Additionally, krill oil naturally contains astaxanthin—a potent carotenoid antioxidant—as well as vitamin A and E derivatives [19,20]. This combination of phospholipid-bound PUFAs and endogenous antioxidants contributes to its superior oxidative stability and anti-inflammatory efficacy [21,22]. Preclinical studies have demonstrated that krill oil attenuates lipid peroxidation, cytokine expression, and fibrosis across hepatic, cardiovascular, renal, and ovarian injury models [5,23,24,25,26,27]. Compared to single-component antioxidants, krill oil provides a multi-targeted cytoprotective mechanism.

Moreover, krill oil offers practical clinical advantages; it is available over the counter, well-tolerated, and is orally administered, making it a cost-effective and accessible candidate for fertility preservation [19,20]. However, its role in protecting against chemotherapy-induced ovarian damage remains underexplored. Therefore, this study aims to investigate whether krill oil can mitigate cisplatin-induced ovarian injury through modulation of oxidative stress and inflammatory pathways.

## 2. Material and Method

### 2.1. Animals

This study was conducted using 21 adult female Wistar albino rats (weighing 200–210 g), which were obtained from an institutional animal research facility. All animals were housed in a standardized environment with controlled ambient temperature (22 ± 2 °C), relative humidity (55 ± 10%), and a 12 h light/dark cycle. Standard pellet chow and tap water were provided ad libitum throughout the experimental period. All experimental procedures were performed in accordance with the ethical standards outlined in the Guide for the Care and Use of Laboratory Animals (NIH, USA), and the study protocol was reviewed and approved by the Institutional Animal Care and Use Committee (IACUC) under ethical approval number 12220409 (approval date: 8 May 2023).

The study was designed and reported in compliance with the ARRIVE (Animal Research: Reporting of In Vivo Experiments) guidelines to ensure transparency and reproducibility. Prior to the experiment, animals were acclimatized to the laboratory conditions for a minimum of one week. All efforts were made to minimize animal suffering and to reduce the number of animals used.

### 2.2. Experimental Protocol

Following a one-week acclimatization period, 21 adult female Wistar rats were randomly divided into three equal groups (*n* = 7 per group). The control group (Group 1) received no pharmacological treatment and was used to establish baseline physiological parameters for all subsequent evaluations.

To induce ovarian toxicity, cisplatin (CP; 2.5 mg/kg, Koçak Pharma, Türkiye) was administered intraperitoneally (i.p.) to the remaining 14 rats twice weekly for four consecutive weeks, resulting in a cumulative dose of 20 mg/kg. After cisplatin initiation, these animals were randomly assigned to two treatment arms:

Group 2 (cisplatin + saline) received 1 mL/kg/day of 0.9% isotonic saline by oral gavage for 28 days. Group 3 (cisplatin + krill oil) was administered krill oil at 4 mL/kg/day by oral gavage for 28 days.

**Figure 1:**
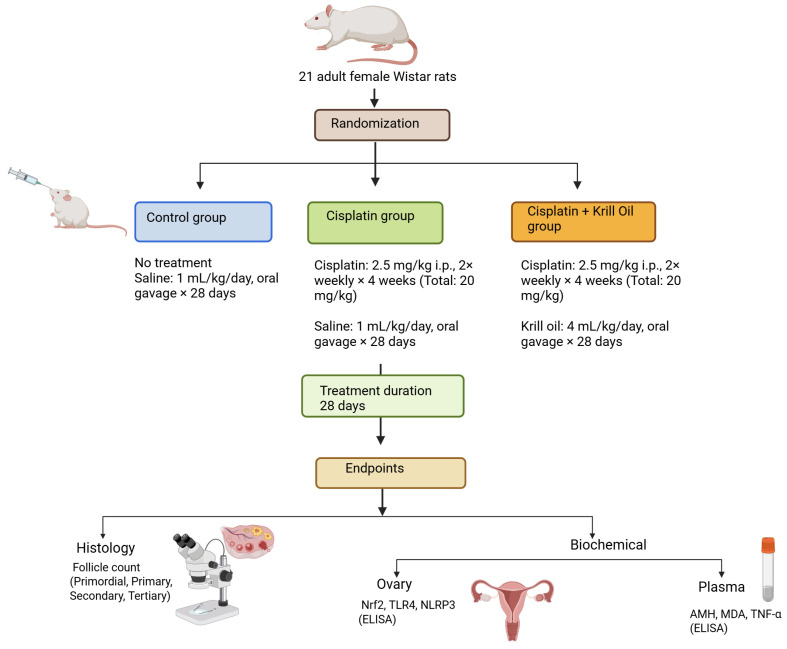
Schematic representation of the experimental design. Adult female Wistar rats were randomized into three groups: control, cisplatin, and cisplatin + krill oil. Cisplatin was administered intraperitoneally (2.5 mg/kg, twice weekly) for 4 weeks to induce ovarian toxicity. Krill oil (4 mL/kg/day) was given by oral gavage for 28 days. Endpoints included histological evaluation of follicle counts and assessment of oxidative stress and inflammatory biomarkers in plasma and ovarian tissues.

At the end of the experimental period, all animals were deeply anesthetized using a combination of ketamine (100 mg/kg; Ketasol^®^, Richterpharma AG, Wels, Austria, EU) and xylazine (50 mg/kg; Rompun^®^, Bayer, Germany) administered intraperitoneally. Blood samples were obtained via cardiac puncture and immediately processed for biochemical analysis. Ovaries were carefully excised and rinsed in ice-cold isotonic saline. For dual downstream analyses, right ovaries were snap-frozen in liquid nitrogen and stored at −80 °C for biochemical assays, while the left ovaries were fixed in formalin for subsequent histological and immunohistochemical analyses. Subsequently, the animals were humanely sacrificed by decapitation. All procedures were conducted in strict accordance with the guidelines (Figure 1).

### 2.3. Histological Examination

At the end of the experimental period, left ovarian tissues were carefully harvested and immediately fixed in 4% neutral-buffered formalin for a minimum of 24 h to ensure optimal preservation of tissue architecture. Following fixation, the samples were processed using a routine histological protocol, which included dehydration in ascending grades of ethanol, clearing in xylene, and embedding in paraffin wax. Serial sections were obtained from each paraffin block at a uniform thickness of 4 µm using a precision rotary microtome (Leica RM2125RT, Leica Biosystems, Nussloch, Germany).

The prepared tissue sections were mounted on poly-L-lysine-coated glass slides, deparaffinized, and stained with hematoxylin and eosin (H&E) for general histomorphological evaluation. Coverslips were applied using a resin-based mounting medium. All stained sections were examined under an Olympus BX51 light microscope equipped with an Olympus C-5050 digital camera system. Representative photomicrographs were captured at 20× magnification from ten randomly selected non-overlapping microscopic fields per section.

Quantitative and qualitative assessments of the ovarian histoarchitecture were performed using a computer-assisted image analysis system (Image-Pro Express v1.4.5; Media Cybernetics, Inc., Rockville, MD, USA). All evaluations were conducted in a blinded fashion by a histopathologist who was unaware of group allocations, ensuring unbiased analysis.

In our study, ovarian follicles were classified into four categories—primordial, primary, secondary, and tertiary follicles—based on established histological criteria [28,29,30].

Primordial follicles were located in the superficial cortex and consisted of a quiescent oocyte surrounded by a single layer of flattened granulosa cells.

Primary follicles contained a centrally placed oocyte encircled by a single layer of cuboidal granulosa cells.

Secondary follicles exhibited multiple concentric layers of cuboidal granulosa cells, with no apparent antral cavity.

Tertiary follicles (antral follicles) displayed a fluid-filled antral cavity, a multilayered stratum granulosum, and well-defined theca interna and externa layers.

Stromal fibrosis was semi-quantitatively assessed based on stromal densification and architectural remodeling visible on hematoxylin–eosin (HE)-stained sections. Fibrotic index was defined as the area of dense, acellular connective stroma in relation to the total ovarian stromal area, calculated by digital image analysis.

### 2.4. Calculation of Percentage Restoration in Follicle Count

In this study, the percentage of recovery was calculated based on the physiological reference values of the control group. This formula demonstrates the extent to which krill oil treatment restores the damage induced by cisplatin toward normal (control) levels. This approach is intended to highlight the restorative effect of the treatment following injury and provides a clearer interpretation of the findings in a clinical context.

Percentage Restoration (%) = [ (Mean of krill oil group − Mean of cisplatin group)/(Mean of control group − Mean of cisplatin group) ] × 100.

### 2.5. Biochemical Analyses

#### 2.5.1. Analysis of Plasma Samples

Plasma samples were subjected to biochemical analyses to evaluate systemic oxidative damage and inflammation.

#### 2.5.2. Measurement of AMH Levels

Blood samples were centrifuged at 3000 rpm for 10 min at room temperature, and the plasma was aliquoted and stored at −20 °C until analysis. Plasma anti-Müllerian hormone (AMH) concentrations were quantified using a commercially available enzyme-linked immunosorbent assay (ELISA) kit (Biosciences, Seattle, WA, USA), following the manufacturer’s protocol. All measurements were performed in duplicate for each sample to ensure assay reliability.

#### 2.5.3. Determination of Lipid Peroxidation

Plasma malondialdehyde (MDA), a major end-product of lipid peroxidation, was quantified using the thiobarbituric acid reactive substances (TBARS) method. Plasma samples were treated with trichloroacetic acid and thiobarbituric acid (TBA) reagent, mixed thoroughly, and incubated at 100 °C for 60 min. Following incubation, samples were cooled on ice and centrifuged at 3000 rpm for 20 min.

The absorbance of the resulting supernatant was measured spectrophotometrically at 535 nm. MDA concentrations were calculated by reference to a standard calibration curve generated using 1,1,3,3-tetraethoxypropane as the external standard. Final results were normalized to total protein content and expressed as nanomoles of MDA per gram of protein (nmol/g protein) [31].

#### 2.5.4. Measurement of TNF-α Levels

Plasma concentrations of tumor necrosis factor-alpha (TNF-α), a major mediator of systemic inflammation, were measured using a rat-specific ELISA kit. The assay was performed according to the manufacturer’s guidelines, with all reagents and samples brought to room temperature prior to use. Absorbance was read at 450 nm using a microplate reader, and cytokine levels were determined from a standard curve and expressed in pg/mL.

#### 2.5.5. Measurement of IL-1β Levels

Plasma IL-1β concentrations were measured using a rat-specific ELISA kit (Rat IL-1β ELISA Kit, Cat. No: E0119Ra, BT Laboratory, Shanghai, China), following the manufacturer’s instructions. The assay was performed using a sandwich ELISA format, and absorbance was recorded at 450 nm. Results were expressed as nanograms per milliliter (ng/mL).

### 2.6. Analysis of Ovarian Tissue Samples

To assess ovarian oxidative stress and inflammatory status, levels of Nrf2, TLR4, NLRP3, IL-1β, and TNF-α were quantified.

#### 2.6.1. Tissue Collection and Storage

Ovaries were excised immediately after sacrifice, rinsed in ice-cold isotonic saline to eliminate residual blood, and stored at −20 °C until biochemical analyses.

#### 2.6.2. Tissue Homogenization

Frozen ovaries were thawed on ice and weighed individually. Each ovary was homogenized in a glass-Teflon homogenizer containing ice-cold phosphate-buffered saline (PBS, pH 7.4) at a ratio of 1:5 (*w*/*v*). Homogenates were centrifuged at 5000× *g* for 15 min at 4 °C, and the clear supernatant was collected and stored at −80 °C for subsequent analysis.

#### 2.6.3. Total Protein Determination

The protein concentration of each supernatant was determined using the Bradford assay [16], with bovine serum albumin (BSA) as the standard. Protein levels were used to normalize all subsequent measurements.

#### 2.6.4. Measurement of Oxidative Stress and Inflammatory Markers

Levels of Nrf2, TLR4, NLRP3, IL-1β, and TNF-α in ovarian tissue were quantified using commercially available rat-specific sandwich ELISA kits according to the manufacturers’ protocols. All samples and reagents were equilibrated to room temperature before use, and measurements were performed in duplicate.

#### 2.6.5. ELISA Data Acquisition and Analysis

Optical density (OD) was measured at 450 nm using a Multiscan GO microplate reader (Thermo Fisher Scientific, Newington, NH, USA). Biomarker concentrations were calculated from the standard curves and normalized to the total protein content, expressed as pg/mg protein.

### 2.7. Statistical Analysis

All statistical analyses were conducted using IBM SPSS Statistics version 19.0 (IBM Corp., Armonk, NY, USA). Normality of data distribution was assessed by the Shapiro–Wilk test, while homogeneity of variances was evaluated using Levene’s test. Parametric data were analyzed by one-way analysis of variance (ANOVA) followed by Tukey’s post hoc test when variances were homogeneous, or Tamhane’s T2 test when homogeneity was violated. For non-parametric data (ovarian fibrosis), the Kruskal–Wallis test was employed, and pairwise comparisons were performed using the Mann–Whitney U test. Data are presented as mean ± standard error of the mean (SEM). A *p*-value < 0.05 was considered statistically significant. All graphical illustrations of group comparisons were generated using GraphPad Prism version 9 (GraphPad Software Inc., San Diego, CA, USA).

## 3. Results

### 3.1. Histopathological Evaluation

#### 3.1.1. Primordial Follicle Count

**Figure 2:**
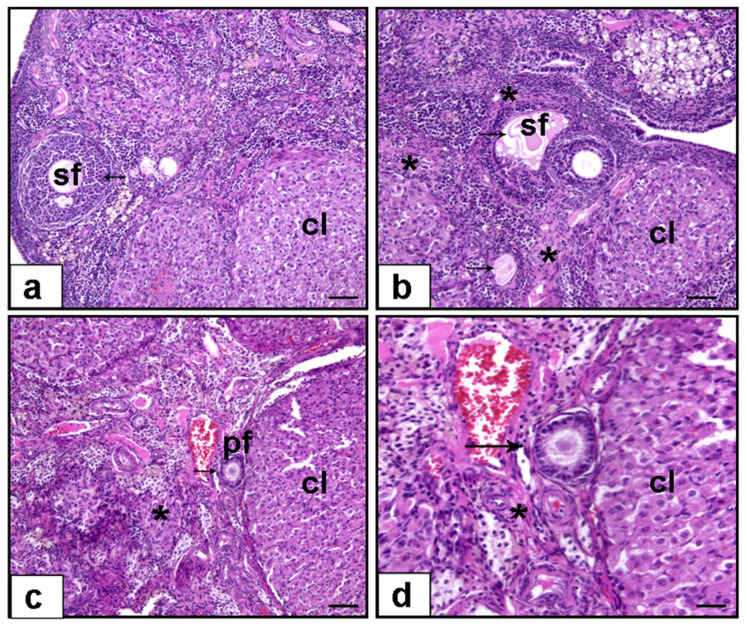
Representative photomicrographs for the light microscopic appearance of ovarian tissues in various groups (hematoxylin and eosin staining). (**a**) Ovarian section from the control group showing normal ovarian architecture and healthy follicles (arrow) (H&E, ×10 magnification). (**b**) Ovarian section from the cisplatin group displaying pronounced stromal fibrosis (asterisk) and follicular atresia (arrow) (H&E, ×10 magnification). (**c**) Ovarian section from the cisplatin + krill oil group demonstrating a marked reduction in stromal fibrosis (asterisk) and preservation of normal follicular structures (arrow) (H&E, ×10 magnification). (**d**) Higher magnification view from the cisplatin + krill oil group highlighting well-preserved ovarian follicles, while residual stromal fibrosis is indicated by an asterisk (*). (H&E, ×40 magnification). Abbreviations: pf, primary follicle; sf, secondary follicle; cl, corpus luteum.

**Figure 3:**
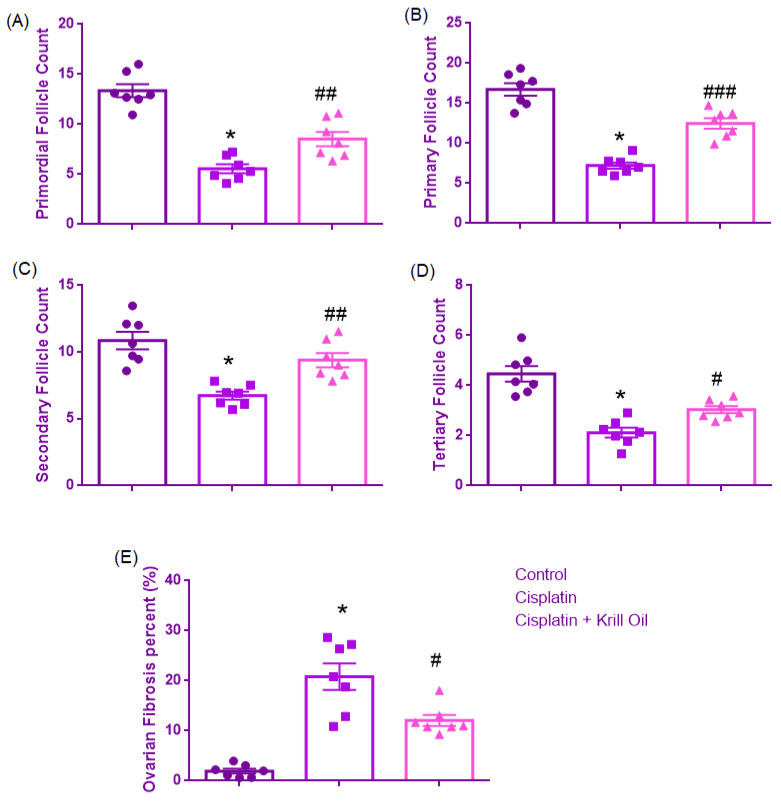
Effect of cisplatin and krill oil co-treatment on ovarian follicle counts and fibrosis. (**A**–**D**) Quantitative comparison of follicle numbers in ovarian tissue: primordial (**A**), primary (**B**), secondary (**C**), and tertiary (**D)** follicles. (**E**) Ovarian fibrosis was expressed as percentage of fibrotic area. Cisplatin significantly reduced the number of all follicle types and increased ovarian fibrosis compared to the control group. Krill oil co-treatment significantly ameliorated these effects, partially restoring follicle counts and attenuating stromal fibrosis. Data are presented as mean ± SEM (n = 7/group). Statistical analysis was performed by one-way ANOVA followed by Tukey’s test for follicle counts and by Mann–Whitney U test for ovarian fibrosis due to violation of normality and variance homogeneity. * *p* < 0.001 different from normal groups; # *p* < 0.05, ## *p* < 0.01, ### *p* < 0.001 different from cisplatin group.

Normality was confirmed for all groups (Shapiro–Wilk, *p* > 0.05), and Levene’s test indicated homogeneity of variances (F(2,18) = 1.024, *p* = 0.379). One-way ANOVA revealed a significant group effect (F(2,18) = 40.503, *p* < 0.001). Primordial follicle counts were significantly reduced in the cisplatin group (5.55 ± 0.4) compared to the control group (13.36 ± 0.6, *p* < 0.001), suggesting early follicular loss due to cytotoxicity. Krill oil co-treatment significantly increased primordial follicle counts relative to the cisplatin group (8.5 ± 0.7, *p* = 0.009), corresponding to a 38.16% improvement compared to the cisplatin group and supporting its role in preserving early follicular development (Figure 2 and Figure 3).

#### 3.1.2. Primary Follicle Count

Normality was established (Shapiro–Wilk, *p* > 0.05), and Levene’s test confirmed homogeneity (F(2,18) = 2.877, *p* = 0.082). ANOVA showed a significant difference among groups (F(2,18) = 56.679, *p* < 0.001). Primary follicle numbers were significantly reduced in the cisplatin group (7.2 ± 0.4) compared to control (16.7 ± 0.7, *p* < 0.001), reflecting impaired follicular maturation. Krill oil treatment significantly improved primary follicle counts compared to cisplatin alone (12.4 ± 0.6, *p* < 0.001), corresponding to a 54.74% improvement compared to the cisplatin group and supporting its role in preserving follicular development at the early maturation stage (Figure 2 and Figure 3).

#### 3.1.3. Secondary Follicle Count

Shapiro–Wilk test confirmed normality (*p* > 0.05), and Levene’s test indicated homogeneity of variances (F(2,18) = 2.718, *p* = 0.093). ANOVA demonstrated significant group differences (F(2,18) = 16.258, *p* < 0.001). Cisplatin administration significantly reduced secondary follicle counts versus control (6.7 ± 0.2 vs. 10.86 ± 0.6, *p* < 0.001), indicating disrupted follicular progression. Krill oil co-treatment significantly elevated secondary follicle numbers compared to the cisplatin group (9.3 ± 0.5, *p* = 0.005), corresponding to a 62.5% improvement compared to the cisplatin group and supporting its role in preserving intermediate-stage follicular development (Figure 2 and Figure 3).

#### 3.1.4. Tertiary Follicle Count

Normality was confirmed (Shapiro–Wilk, *p* > 0.05), and Levene’s test showed no significant variance difference (F(2,18) = 2.570, *p* = 0.104). A significant group effect was found by ANOVA (F(2,18) = 26.757, *p* < 0.001). Tertiary follicle numbers were markedly decreased in the cisplatin group relative to control (2.1 ± 0.19 vs. 4.4 ± 0.3, *p* < 0.001), indicating advanced follicle degeneration. Krill oil administration significantly restored tertiary follicle development compared to the cisplatin group (3.03 ± 0.1, *p* = 0.029), corresponding to a 40.43% improvement compared to the cisplatin group and supporting its role in preserving late-stage follicular development (Figure 2 and Figure 3).

#### 3.1.5. Ovarian Fibrosis (%)

Normality assumption was not met in the krill oil group (Shapiro–Wilk, *p* = 0.041). Therefore, a non-parametric approach was adopted. The Kruskal–Wallis test showed a significant difference among the groups (*p* < 0.001). Pairwise comparisons using the Mann–Whitney U test revealed that ovarian fibrosis percentage was significantly increased in the cisplatin group (20.81 ± 2.67%) compared to the control group (1.93 ± 0.4%, *p* = 0.001), indicating marked stromal injury and extracellular matrix deposition. Krill oil co-treatment significantly decreased ovarian fibrosis levels compared to cisplatin alone (12.08 ± 1.08%, *p* = 0.017), suggesting an antifibrotic effect potentially mediated by inhibition of inflammation-driven fibroblast activation and oxidative stress-induced collagen production (Figure 2 and Figure 3).

### 3.2. Assessment of Biochemical Parameters

#### 3.2.1. Plasma AMH (ng/mL) Level

**Figure 4:**
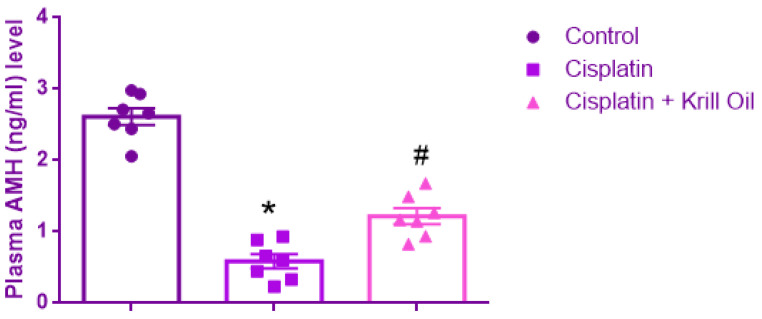
Effect of cisplatin and krill oil co-treatment on plasma AMH levels. Data are expressed as mean ± SEM (n = 7 per group). Statistical analysis was performed using one-way ANOVA followed by appropriate post hoc tests. * *p* < 0.001 different from normal groups; # *p* < 0.01 different from cisplatin group.

Normality was confirmed for all groups (Shapiro–Wilk, *p* > 0.05), and Levene’s test indicated homogeneity of variances (F(2,18) = 0.039, *p* = 0.962). One-way ANOVA revealed a significant group effect (F(2,18) = 87.814, *p* < 0.001). AMH levels were significantly decreased in the cisplatin group (0.58 ± 0.1 ng/mL) compared to control (2.6 ± 0.11 ng/mL, *p* < 0.001), indicating cisplatin-induced gonadotoxicity and follicular damage. Krill oil co-treatment significantly increased AMH levels compared to the cisplatin group (1.21 ± 0.1 ng/mL, *p* = 0.002), suggesting a protective effect on ovarian reserve through its antioxidant and anti-inflammatory mechanisms (Figure 4).

#### 3.2.2. Plasma MDA and Nrf2 (Oxidative Stress)

##### Plasma MDA Level (nmol/g)

**Figure 5:**
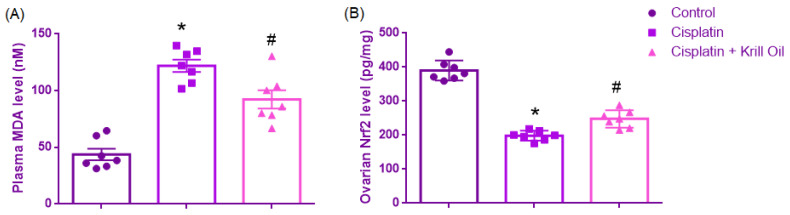
Effect of cisplatin and krill oil co-treatment on plasma MDA (**A**) and ovarian Nrf2 (**B**) levels. Data are expressed as mean ± SEM (n = 7 per group). Statistical analysis was performed using one-way ANOVA followed by appropriate post hoc tests. * *p* < 0.001 different from normal groups; # *p* < 0.01 different from cisplatin group.

Normality assumption was met (Shapiro–Wilk, *p* > 0.05), and Levene’s test confirmed homogeneity (F(2,18) = 0.921, *p* = 0.416). ANOVA results showed a significant group effect (F(2,18) = 39.298, *p* < 0.001). MDA levels were significantly elevated in the cisplatin group (122 ± 5.4 nM) compared to control (43.76 ± 5.0 nM, *p* < 0.001), reflecting increased lipid peroxidation and oxidative stress. Krill oil significantly reduced MDA levels compared to cisplatin (92.30 ± 7.9 nM, *p* = 0.009), indicating attenuation of oxidative damage and restoration of redox balance (Figure 5).

##### Ovarian NrF2 Level (pg/mg)

Normal distribution was confirmed (Shapiro–Wilk, *p* > 0.05), and Levene’s test indicated homogeneity (F(2,18) = 1.639, *p* = 0.221). A significant difference among groups was observed (F(2,18) = 119.390, *p* < 0.001). Nrf2 levels were significantly reduced in the cisplatin group compared to control (198.5 ± 5.4 vs. 390.3 ± 11.2 pg/mg, *p* < 0.001), reflecting impaired antioxidant defense. Krill oil co-treatment significantly restored Nrf2 expression (248 ± 9.6 pg/mg, *p* = 0.003), supporting its role in enhancing endogenous antioxidant pathways and mitigating oxidative stress (Figure 5).

#### 3.2.3. Ovarian NLRP3 and IL-1β + Plasma IL-1β

##### Ovarian NLRP-3 Level (pg/mg)

**Figure 6:**
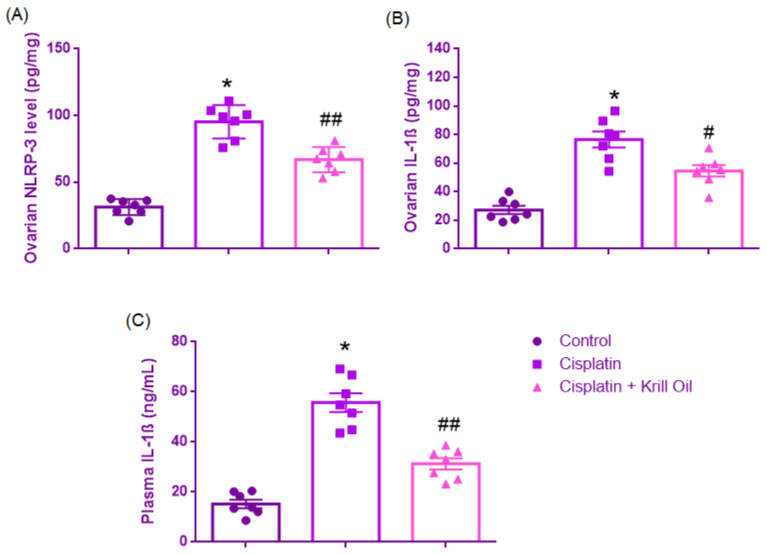
Effect of cisplatin and krill oil co-treatment on ovarian NLRP3 (**A**), ovarian IL-1β (**B**), and plasma IL-1β levels (**C**). Data are presented as mean ± SEM (n = 7 per group). Statistical analysis was performed using one-way ANOVA followed by appropriate post hoc tests. * *p* < 0.001 different from normal groups; # *p* < 0.01, ## *p* < 0.001 different from cisplatin group.

Normality was established (Shapiro–Wilk, *p* > 0.05), and Levene’s test verified equal variances (F(2,18) = 1.411, *p* = 0.270). One-way ANOVA revealed significant group differences (F(2,18) = 76.437, *p* < 0.001). NLRP3 levels were significantly increased in the cisplatin group compared to control (95.31 ± 4.73 vs. 31.29 ± 2.26 pg/mg, *p* < 0.001), indicating inflammasome activation and sterile inflammation. Krill oil treatment significantly downregulated NLRP3 expression compared to cisplatin (66.97 ± 3.58 pg/mg, *p* < 0.001), suggesting suppression of inflammasome-mediated injury and innate immune modulation (Figure 6).

##### Ovarian IL-1β Level (pg/mg)

Normal distribution was confirmed across groups (Shapiro–Wilk test, *p* > 0.05), and homogeneity of variances was supported by Levene’s test (F(2,18) = 1.222, *p* = 0.318). One-way ANOVA revealed a significant effect of treatment on ovarian IL-1β levels (F(2,18) = 33.198, *p* < 0.001). Post hoc comparisons using Tukey’s HSD test demonstrated that cisplatin administration markedly elevated ovarian IL-1β levels compared to the control group (76.54 ± 5.53 vs. 27.25 ± 2.94 pg/mg, *p* < 0.001), indicating a robust pro-inflammatory response. Co-administration of krill oil significantly reduced IL-1β expression relative to cisplatin alone (54.58 ± 3.99 pg/mg, *p* = 0.005), suggesting partial mitigation of inflammation by krill oil (Figure 6).

##### Plasma IL-1β Level (ng/mL)

Shapiro–Wilk test confirmed the normality of distribution (*p* > 0.05) and Levene’s test indicated that variances were homogeneous (F(2,18) = 3.008, *p* = 0.075). One-way ANOVA revealed a significant group effect on plasma IL-1β levels (F(2,18) = 55.781, *p* < 0.001). Post hoc comparisons using Tukey’s HSD test showed that cisplatin administration significantly elevated IL-1β concentrations compared to the control group (55.67 ± 3.78 vs. 15.27 ± 1.69 ng/mL, *p* < 0.001), indicating a pronounced systemic inflammatory response. Co-treatment with krill oil significantly reduced plasma IL-1β levels compared to the cisplatin group (31.32 ± 2.26 ng/mL, *p* < 0.001), suggesting partial systemic anti-inflammatory activity (Figure 6).

#### 3.2.4. Ovarian TLR4 and TNF-α + Plasma TNF-α

##### Ovarian TLR4 Level (ng/mg)

**Figure 7:**
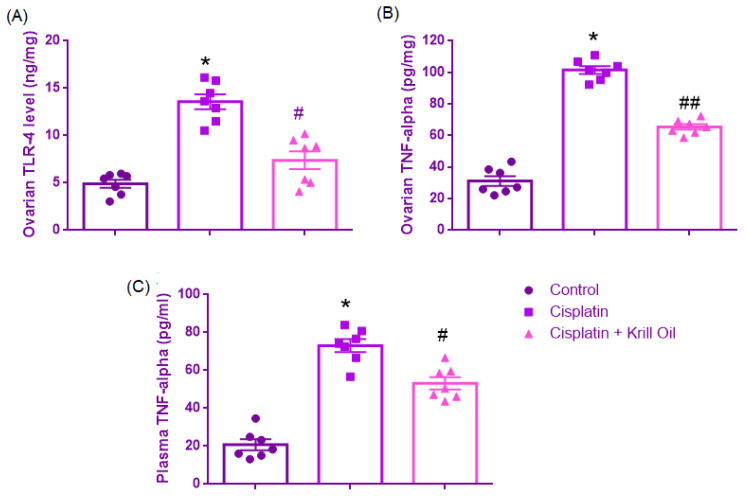
Effect of cisplatin and krill oil co-treatment on ovarian tissue TLR4 (**A**), TNF-α (**B**), and plasma TNF-α (**C**) levels. Data are presented as mean ± SEM (n = 7 per group). Statistical analysis was performed using one-way ANOVA followed by appropriate post hoc tests. * *p* < 0.001 different from normal groups; # *p* < 0.01, ## *p* < 0.001 different from cisplatin group.

Normality was confirmed (Shapiro–Wilk, *p* > 0.05), and Levene’s test indicated heterogeneity of variances (F(2,18) = 4.144, *p* = 0.033). Therefore, post hoc comparisons were conducted using Tamhane’s T2 test. One-way ANOVA revealed a significant group effect (F(2,18) = 35.459, *p* < 0.001). TLR4 levels were significantly increased in the cisplatin group compared to control (13.57 ± 0.79 vs. 4.9 ± 0.43 ng/mg, *p* < 0.001), suggesting innate immune activation through TLR4 signaling. Krill oil co-treatment significantly lowered TLR4 expression relative to the cisplatin group (7.38 ± 0.93 ng/mg, *p* = 0.001), indicating attenuation of pro-inflammatory receptor signaling (Figure 7).

##### Ovarian TNF-α Levels (pg/mg)

Normal distribution was confirmed across all groups (Shapiro–Wilk test, *p* > 0.05), and Levene’s test indicated homogeneity of variances (F(2,18) = 2.314, *p* = 0.128). One-way ANOVA revealed a statistically significant main effect of treatment on ovarian TNF-α levels (F(2,18) = 200.172, *p* < 0.001). Post hoc analysis using Tukey’s HSD test demonstrated that TNF-α concentrations were significantly elevated in the cisplatin group compared to the control group (101.5 ± 2.44 vs. 31.20 ± 3.08 pg/mg, *p* < 0.001), suggesting an intense pro-inflammatory response induced by cisplatin. Notably, krill oil co-administration significantly attenuated this elevation (65.47 ± 1.76 pg/mg, *p* < 0.001 vs. cisplatin), indicating partial anti-inflammatory efficacy (Figure 7).

##### Plasma TNF-α (pg/mL)

Shapiro–Wilk test confirmed normality (*p* > 0.05), and Levene’s test indicated equal variances (F(2,18) = 0.174, *p* = 0.842). A significant group effect was observed (F(2,18) = 68.996, *p* < 0.001). TNF-α levels were significantly increased in the cisplatin group versus control (73.06 ± 3.4 vs. 20.79 ± 2.8 pg/mL, *p* < 0.001), indicating inflammatory cytokine activation. Krill oil co-treatment significantly reduced TNF-α levels compared to the cisplatin group (53.17 ± 3.2 pg/mL, *p* = 0.001), suggesting suppression of inflammatory cascades (Figure 7).

## 4. Discussion

In this study, we demonstrated that krill oil supplementation significantly mitigated cisplatin-induced ovarian damage in rats. Histological evaluation showed preservation of ovarian follicle populations and reduction in stromal fibrosis, while biochemical analyses confirmed restoration of oxidative balance and suppression of inflammatory pathways. These findings suggest that krill oil exerts protective effects on ovarian reserve by targeting multiple pathological mechanisms involved in chemotherapy-induced ovarian injury.

Cisplatin, a widely used chemotherapeutic agent, is known to accelerate follicular depletion, thereby increasing the risk of premature ovarian failure (POF) and infertility in premenopausal women [2,3,32,33,34]. Quantitative assessment of follicle populations remains the gold standard for evaluating reproductive potential. Consistent with prior studies [5,6,35,36], our findings revealed that cisplatin exposure significantly depleted primordial and growing follicles (primary, secondary, and tertiary), leading to widespread ovarian injury. Importantly, krill oil supplementation markedly ameliorated histological damage, as evidenced by reduced fibrosis and preserved follicular integrity, aligning with earlier reports of krill oil improving follicular counts and attenuating histological alterations in ovarian ischemia–reperfusion models [27]. The ability of krill oil to preserve follicle populations across developmental stages may reflect its dual regulation of redox homeostasis and immune-mediated injury.

Anti-Müllerian hormone (AMH), secreted by granulosa cells of small antral follicles, serves as a specific and sensitive biomarker of ovarian reserve by inhibiting primordial follicle recruitment and reducing FSH sensitivity [37,38,39]. Previous studies have demonstrated that AMH levels decline following cisplatin-induced ovarian damage, reflecting depletion of the follicular pool [9,35,36,40]. Consistent with these findings, our study revealed a significant reduction in plasma AMH concentrations after cisplatin administration, indicating substantial impairment of ovarian reserve. This reduction is likely attributable to granulosa cell apoptosis induced by cisplatin, which suppresses AMH production and leads to follicular loss [36,41]. Furthermore, recent studies have demonstrated that cisplatin induces overactivation of dormant primordial follicles through the PI3K/Akt/mTORC1 signaling pathway, leading to follicular atresia, AMH decline, and accelerated depletion of the follicular pool [42]. Notably, krill oil supplementation effectively counteracted these effects by preserving primordial follicle counts and restoring AMH levels, suggesting a protective role in maintaining granulosa cell integrity and AMH expression.

The underlying mechanism of cisplatin-induced ovarian toxicity involves excessive reactive oxygen species (ROS) generation and impaired antioxidant defenses. Previous studies demonstrated that cisplatin elevates oxidative stress markers such as malondialdehyde (MDA) while suppressing the Nrf2 pathway, a key regulator of antioxidant gene expression [8,9], thereby promoting lipid peroxidation, mitochondrial dysfunction, and granulosa cell apoptosis. In our study, krill oil treatment restored Nrf2 expression and reduced MDA levels, consistent with its known antioxidant activity.

Several antioxidant agents, including melatonin, rutin, chrysin, rosmarinic acid, resveratrol, and mesna, have demonstrated protective effects against cisplatin-induced ovarian injury by attenuating oxidative stress [5,13,33,36,43,44,45,46]. Similarly, in the present study krill oil supplementation restored Nrf2 expression and decreased MDA levels, indicating effective mitigation of oxidative ovarian injury. The protective properties of krill oil are likely attributable to its bioactive components, particularly omega-3 polyunsaturated fatty acids (PUFAs; EPA and DHA) and astaxanthin [47]. Astaxanthin stabilizes Nrf2 by inhibiting Keap1 binding, promoting its nuclear translocation and transcription of antioxidant genes [23,48]. Supporting these findings, Kükürt and Karapehlivan [25] demonstrated that astaxanthin reduced oxidative damage and enhanced antioxidant enzyme activity in rat ovaries, while Yang et al. [49] reported that astaxanthin upregulated Nrf2 expression in porcine granulosa cells. Additionally, Nair et al. [50] demonstrated that omega-3 supplementation restored antioxidant defenses in ovarian toxicity models. Together, these data suggest that krill oil protects against oxidative ovarian damage by limiting lipid peroxidation and promoting Nrf2-mediated cytoprotection.

In cisplatin-induced ovarian injury, not only oxidative stress but also a strong TLR4-mediated inflammatory response plays a significant role. TLR4 can be directly activated by cisplatin in an endotoxin-independent manner, initiating downstream signaling through MyD88 to activate the NF-κB and MAPK pathways, thereby increasing the expression of pro-inflammatory mediators such as TNF-α, IL-6, and IL-1β [41,51,52,53]. Following this early response, NLRP3 inflammasome activation occurs in the late phase of inflammation. Cisplatin-induced oxidative stress and ATP release provide secondary stimuli that trigger inflammasome assembly, promoting caspase-1-mediated conversion of pro-IL-1β into its mature form [47,54,55]. In line with these mechanisms, our data showed that cisplatin significantly upregulated ovarian TLR4 and NLRP3 protein levels, along with elevated TNF-α and IL-1β concentrations in both ovarian and plasma samples.

Recent in vitro and in vivo studies have demonstrated that krill oil exerts anti-inflammatory effects primarily through modulation of TLR4-dependent signaling pathways. Krill oil has been shown to suppress LPS-induced TNF-α and IL-1β production by inhibiting the TLR4/NF-κB/NLRP3 signaling cascade in diverse inflammatory models [6,56,57,58]. These findings align with our results, in which krill oil treatment markedly attenuated both early (TLR4/TNF-α) and late (NLRP3/IL-1β) inflammatory responses, thereby protecting ovarian tissue from cisplatin-induced injury. The observed reductions in ovarian NLRP3 and IL-1β protein levels, along with decreased TNF-α and IL-1β levels in plasma, suggest effective suppression of inflammasome activation.

These anti-inflammatory effects are attributed to krill oil’s bioactive constituents. Omega-3 fatty acids, particularly EPA and DHA, modulate membrane lipid composition, inhibit TLR4/NF-κB signaling, and reduce arachidonic acid–derived eicosanoid production [59,60,61]. Astaxanthin, another key component of krill oil, limits NF-κB nuclear translocation in macrophages, thereby suppressing IL-6 and TNF-α secretion and reducing inflammatory tissue damage [6,24,62]. Taken together, our findings indicate that krill oil modulates immune signaling by targeting both upstream (TLR4/TNF-α) and downstream (NLRP3/IL-1β) inflammatory pathways, contributing to its anti-inflammatory efficacy.

Cisplatin-induced ovarian injury is also characterized by stromal fibrosis due to excessive collagen deposition, which disrupts the ovarian microenvironment and impairs folliculogenesis [12]. Since both oxidative stress and inflammation drive fibrotic remodeling, krill oil may preserve stromal architecture by limiting fibroblast activation and collagen accumulation. Suppression of NLRP3 inflammasome activity may further contribute to reduced fibrogenesis [12,58].

The clinical feasibility of krill oil as a fertility-protective adjunct during chemotherapy is supported by its favorable pharmacological and practical properties. Krill oil is administered orally and is readily absorbed; its phospholipid-bound omega-3s achieve efficient systemic levels without requiring high doses [18]. It is generally regarded as safe and well-tolerated, with minor side effects similar to fish oil and no serious toxicity reported in human studies [23,63]. As an over-the-counter supplement, krill oil provides a more accessible and cost-effective option compared to invasive fertility preservation strategies. Unlike GnRHa co-therapy—which may cause hypoestrogenic symptoms and is often expensive and logistically complex [64]—krill oil avoids hormonal suppression and can be easily integrated into treatment regimens. Although clinical trials on krill oil’s direct role in fertility preservation are lacking, its benefit is supported by evidence from human studies on PMS and dysmenorrhea, where it reduced symptom severity and analgesic need more effectively than fish oil [65,66]. Its anti-inflammatory effects in arthritis and cardiovascular patients further underscore its safety and broad applicability [67,68,69,70]. Taken together, these results highlight krill oil’s capacity to counteract the multifactorial nature of chemotherapy-induced ovarian injury through integrated antioxidant, anti-inflammatory, and antifibrotic actions.

This study has some limitations. Long-term reproductive outcomes, including estrous cyclicity and fertility, were not evaluated. Moreover, only one chemotherapeutic agent and dosage were assessed in a single animal model. Future studies should explore the effects of krill oil across different chemotherapy regimens and incorporate functional reproductive endpoints. Translation to clinical settings will require validation in human studies.

A major limitation of this study is the evaluation of ovarian fibrosis based solely on routine hematoxylin and eosin (H&E) staining. Although structural alterations were observed, the use of collagen-specific histochemical stains such as Masson’s trichrome or Picrosirius red would have provided a more precise assessment of fibrotic changes. Future studies should incorporate these staining methods to better characterize collagen deposition and fibrosis severity.

## 5. Conclusions

Our study is among the first to demonstrate the dual antioxidant and anti-inflammatory effects of krill oil in a model of chemotherapy-induced ovarian toxicity. Krill oil preserved follicular integrity, limited stromal fibrosis, reduced oxidative stress, and restored Nrf2-dependent antioxidant defenses, leading to the maintenance of AMH levels. Moreover, it suppressed TLR4/TNF-α/NLRP3/IL-1β signaling pathways, thereby alleviating ovarian inflammation. Collectively, these findings suggest that krill oil exerts its protective effects through coordinated antioxidant and anti-inflammatory mechanisms, offering a promising strategy for preserving ovarian reserve during chemotherapy.

## Data Availability

The data presented in this study are available on request from the corresponding author.
